# Reduced Graphene Oxide-Wrapped Novel CoIn_2_S_4_ Spinel Composite Anode Materials for Li-ion Batteries

**DOI:** 10.3390/nano12244367

**Published:** 2022-12-07

**Authors:** Ting-Yu Lee, Wei-Ren Liu

**Affiliations:** Department of Chemical Engineering, R&D Center for Membrane Technology, Center for Circular Economy, Chung Yuan Christian University, 200 Chung Pei Road, Chung Li District, Taoyuan City 32023, Taiwan

**Keywords:** transition metal sulfides, reduced graphene oxide, lithium-ion battery, CoIn_2_S_4_, anode

## Abstract

In this study, we proposed a novel CoIn_2_S_4_/reduced graphene oxide (CoIn_2_S_4_/rGO) composite anode using a hydrothermal method. By introducing electronic-conductive reduced graphene oxide (rGO) to buffer the extreme volume expansion of CoIn_2_S_4_, we prevented its polysulfide dissolution during the lithiation/de-lithiation processes. After 100 cycles, the pristine CoIn_2_S_4_ electrode demonstrated poor cycle performance of only 120 mAh/g at a current density of 0.1 A/g. However, the composition-optimized CoIn_2_S_4_/rGO composite anode demonstrated a reversible capacity of 580 mAh/g for 100 cycles, which was an improvement of 4.83 times. In addition, the ex situ XRD measurements of the CoIn_2_S_4_/rGO electrode were conducted to determine the reaction mechanism and electrochemical behavior. These results suggest that the as-synthesized CoIn_2_S_4_/rGO composite anode is a promising anode material for lithium ion batteries.

## 1. Introduction

Technological development and economic growth have led to an increase in energy demands. As a renewable energy, lithium-ion batteries (LIBs) are regarded as the most promising energy storage and conversion materials [1,2]. Their long cycle life and high energy density have afforded them widespread adoption and applications in various areas, including electric vehicles and portable electronic devices [3,4,5]. Graphite is the current commercially available material for LIBs. However, the energy and power density of graphite anodes are insufficient for electric-powered vehicles [6,7]. To replace graphite, scientists are urgently developing novel materials with greater reversible capacity.

According to the storage mechanisms of lithium ions, anode materials are categorized as intercalation based, alloy based, and conversion based. Among these anodes, conversion anodes have garnered great interest due to their high theoretical capacity and remarkable stability for lithium storage. Transition metal sulfides (TMSs), such as Co_9_S_8_ [8,9,10], Co_3_S_4_ [11,12], CoS [13,14,15], CuS [16,17,18], and NiS [19,20], are some of the most well-known conversion-based anodes because they are inexpensive and environmentally friendly. Compared to the preceding metal sulfides, bimetallic sulfides have superior electrochemical performance. Bimetallic sulfides can provide sufficient active sites and have a higher specific capacity than sulfides composed of a single metal. For bimetallic sulfides, the characteristics of spinel-based metal sulfides have garnered considerable interest because they can result in greater ionic conductivity [21,22,23,24,25]. Verma et al. [26] successfully synthesized carbon-coated CuCo_2_S_4_ anode materials for LIBs using a straightforward in situ hydrothermal method. A composition-optimized CuCo_2_S_4_/C composite anode exhibited 361 mAh/g of reversible capacity after 30 cycles at 0.2C current density. In contrast, the CuCo_2_S_4_ anode’s reversible capacity after 30 cycles at the same current density was only 175 mAh/g. Introducing in situ carbon-coating technology decreased the SEI film resistance and charge transfer resistance, as demonstrated by a Nyquist plot. By altering the solvent ratio of EG (ethylene glycol)/TBA (tert-butyl alcohol), Li’s group was able to prevent the surface morphologies of CuCo_2_S_4_/rGO from aggregating [27]. Well-dispersed CuCo_2_S_4_/rGO had a particle size distribution of sub-microspheres in the range of 0.3–0.4 μm, which could significantly improve its cycle stability. CuCo_2_S_4_/rGO demonstrated high reversible capacities of 1049, 998, 932, 801 and 751 mAh/g at different current densities of 0.1, 0.2, 0.5, 1 and 2 A/g, respectively. While the current density returned to 0.1 A/g, the CuCo_2_S_4_/rGO battery delivered 1035 mAh/g with a capacity retention of over 98%. Zhang’s research group reported the fabrication of ZnIn_2_S_4_ and spray-dried graphene oxide (GO) to synthesize a ZnIn_2_S_4_@GO composite anode for LIBs [28]. The surface morphology of the as-synthesized ZnIn_2_S_4_ anode exhibited a 3D flower-like structure that offered nanochannels for transporting Li-ions during the lithiation and de-lithiation processes. The ZnIn_2_S_4_@GO composite exhibited a lower charge transfer resistance than the ZnIn_2_S_4_ anode, indicating that incorporating a graphene-based carbon matrix facilitated a faster interfacial charge transfer, as evidenced by the Nyquist plot. Even after 200 cycles, ZnIn_2_S_4_@GO exhibited a higher capacity of 476 mAh/g than pristine ZnIn_2_S_4_. By providing multiple Li^+^ transport channels and dramatically buffering volume expansion during lithiation/de-lithiation processes, the GO coating significantly contributed to the development of the electrochemical exhibition.

In our previous research, we successfully utilized a hydrothermal method and a ball-milling process to synthesize bare CuIn_2_S_4_ and CuIn_2_S_4_/C composites and investigated the corresponding reaction mechanism using ex situ XRD measurements. For CuIn_2_S_4_ and CuIn_2_S_4_/C, the diffusivity of Li ions was 2.58 × 10^−15^ and 1.11 × 10^−12^ cm^2^∙s^−1^, respectively. The results suggested that introducing carbon as a conductive matrix could enhance the reversible capacity and rate capability of LIBs [29]. Cu is an inactive material as an anode for Li ion batteries. Thus, Cu cannot contribute any reversible capacity during charge and discharge processes. That is the reason why we chose Co to replace Cu in the spinel structure. In addition, no literature focuses on a CoIn_2_S_4_/rGO composite anode for Li ion batteries. Thus, in this study, we first synthesized CoIn_2_S_4_ using a hydrothermal method as an anode material for LIBs. In contrast, as-synthesized CoIn_2_S_4_ was wrapped in graphene oxide, which prevents CoIn_2_S_4_ aggregation [30]. The corresponding characterizations, including XRD (X-ray diffraction), SEM (scanning electron microscope), HRTEM (high-resolution transmission electron microscope), EDX (energy-dispersive X-ray), XPS (X-ray photoelectron spectroscopy), and BET (Brunauer–Emmett–Teller method) isotherms, were carried out to determine the crystal structure, surface morphology, elemental distributions, charge states, specific surface area, and pore size distribution, respectively. Ex situ XRD analysis was utilized to investigate and demonstrate the corresponding reaction mechanism of the condition-optimized CoIn_2_S_4_/C composite anode. Our method of incorporating GO improved electron transport and ion diffusion, providing a viable structure for high-performance LIBs.

## 2. Experimental Section

### 2.1. Synthesis of Pristine CoIn_2_S_4_ and CoIn_2_S_4_/rGO Composite

**Scheme 1:**
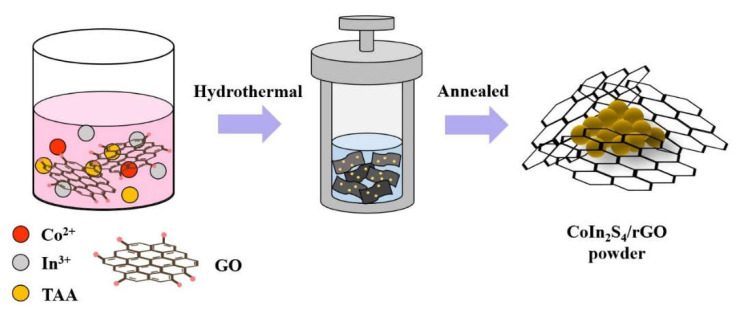
Preparation process of CuIn_2_S_4_/C composite.

The Hummers’ technique was adapted to synthesize GO [31]. CoIn_2_S_4_/rGO was prepared via a simple hydrothermal process. Initially, GO powder was ultrasonically dispersed in 50 mL of deionized (DI) water for 30 min. Subsequently, 1 mmol of Co(NO_3_)_2_·6H_2_O (Cobalt(II) nitrate, 98%, SHOWA), 2 mmol of In(NO_3_)_3_ (Indium nitrate, 99.99%, Alfa Aesar, MA, USA), and 8 mmol of CH_3_CSNH_2_ (Thioacetamide, 99%, Alfa Aesar, Haverhill) were added to the above solution and stirred for 2 h. Then 15 mmol of CH_4_N_2_O (Urea, 99%, Sigma Aldrich, St. Louis, MO, USA) was added dropwise. After vigorously stirring the mixture for 1 h, it was placed in a 100 mL Teflon-lined stainless steel autoclave and heated at 180 °C for 24 h. The CoIn_2_S_4_ powder was obtained by high-speed centrifugation and multiple washes with DI water and ethanol at room temperature before natural cooling. The samples were then vacuum-dried at 70 °C for 12 h. In contrast, pristine CoIn_2_S_4_ was synthesized via a similar procedure, excluding the addition of GO to the mixture solution (Scheme 1).

### 2.2. Characterizations

The crystal structure of the as-prepared sample was confirmed by X-ray diffraction (XRD, Bruker D2 phaser). The morphology of the samples was analyzed using a scanning electron microscope (SEM, JEOL JSM-7600M) equipped with energy-dispersive X-ray spectroscopy (EDX) and a high-resolution transmission electron microscope (HRTEM, Hitachi, H-7100). X-ray photoelectron spectroscopy (XPS, Thermo Fisher Scientific, Waltham, MA, USA) was utilized to determine the elementary binding state and valences. Using the Brunauer–Emmett–Teller (BET) method based on the N_2_ adsorption-desorption isotherms obtained with a Micromeritics Tristar 3000, the specific surface area of the samples was determined.

### 2.3. Electrochemical Measurements

Active materials, conductive additive (Super-P), and binder (polyvinylidene fluoride, PVDF) were mixed at a weight ratio of 8:1:1 in NMP solvent (N-methyl-2-pyrrolidone) to form the working electrode. This slurry was then used to coat a 10 µm thick copper foil current collector. The electrode was punched into a 14 mm diameter circle before being vacuum-dried at 120 °C for 8 h. The coin-type half cells (CR2032) were assembled in an argon-filled glovebox. Here, less than 0.5 ppm of oxygen and moisture levels must be maintained. Next, 1M LiPF_6_ was dissolved in a 1:1:1 wt% solution of ethylene carbonate, ethyl methyl carbonate, and dimethyl carbonate (EC:EMC:DMC). The separator we used was a 25 μm trilayer Celgard 2325. The counter was lithium foil with a thickness of 200 μm. The coin cells (CR2032) were tested in the potential range of 0.01–3.0 V at various current densities to determine their stability. The electrochemical workstation (CH Instruments Analyzer CHI 6273E) with a potential range of 0.01–3.0 V and a scan rate of 0.1 mV/s was used to test the cyclic voltammetry (CV) investigations. The frequency ranges for electrochemical impedance spectroscopy (EIS) were 1 MHz–10 mHz.

## 3. Results and Discussion

**Figure 1:**
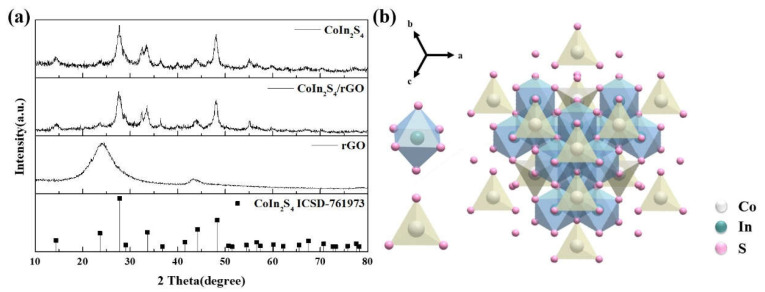
(**a**) XRD patterns of CoIn_2_S_4,_ CoIn_2_S_4_/rGO, and rGO samples; (**b**) crystal structure of CoIn_2_S_4_.

The XRD patterns of bare CoIn_2_S_4_ and CoIn_2_S_4_/rGO composites (Figure 1a) were tested from 10 to 80°. All the diffraction peaks were consistent with CoIn_2_S_4_ (ICSD 76-1973). In addition, the diffraction peaks of CoIn_2_S_4_/rGO at 28, 34, 44, and 48° were attributed to the (311), (400), (511), and (440) crystal planes of CoIn_2_S_4_. Figure 1b depicts the crystal structure of CoIn_2_S_4_. The CoIn_2_S_4_ possessed a cubic crystal structure with the space group $Fd\bar{3}m$. Cobalt cations were placed in tetrahedral 8a sites, indium cations in octahedral 16d sites, and sulfur anions in tetrahedral 32e sites to form the corner-sharing tetrahedral network.

**Figure 2:**
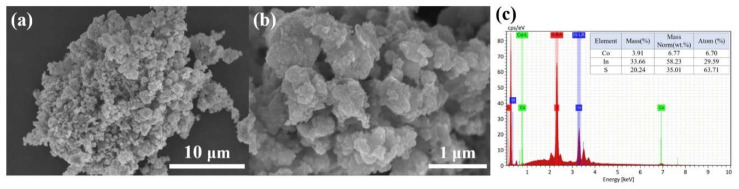
(**a**,**b**) SEM images of CoIn_2_S_4_; (**c**) EDS spectrum of CoIn_2_S_4_.

SEM and HRTEM were used to examine the surface morphologies of the as-prepared samples. As depicted in Figure 2a,b, the as-synthesized CoIn_2_S_4_ exhibited an irregular morphology, a relatively smooth surface, and inhomogeneous diameters of about 1–2 μm. The EDS mapping of CoIn_2_S_4_ is shown in Figure 2c. The computed molar concentrations of Co, In, and S in CoIn_2_S_4_ were 6.70, 29.59, and 63.71%, respectively.

**Figure 3:**
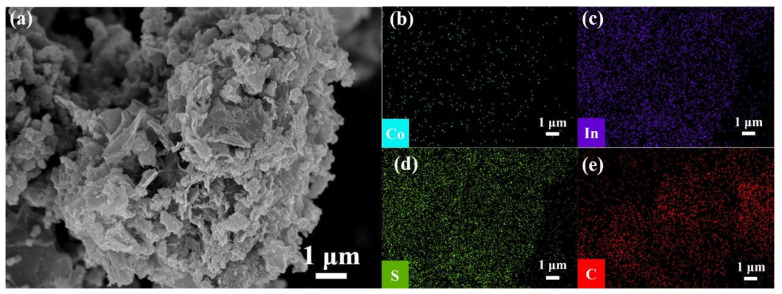
(**a**) SEM image of CoIn_2_S_4_/rGO; EDX mapping of (**b**) Co, (**c**) In, (**d**) S and (**e**) C.

**Figure 4:**
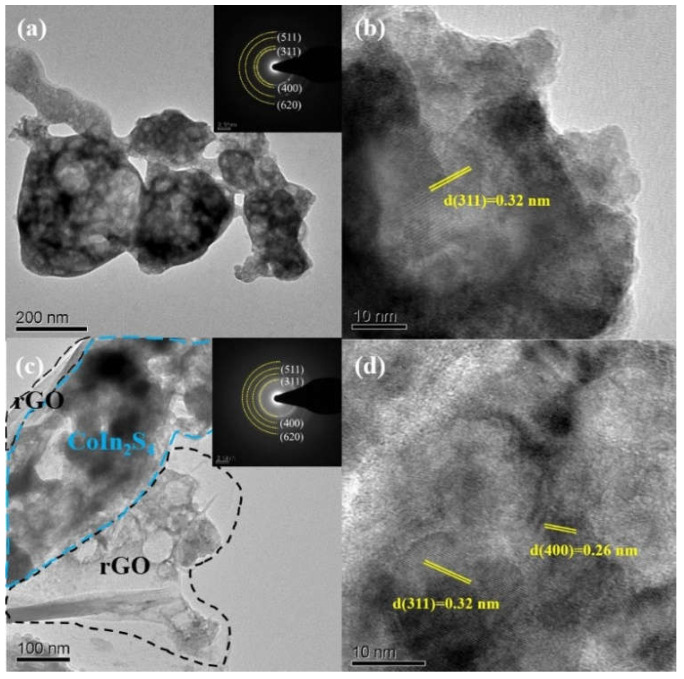
HR-TEM images with SAED pattern inset (selected area electron diffraction) of (**a**,**b**) CoIn_2_S_4_ and (**c**,**d**) CoIn_2_S_4_/rGO.

In addition, the SEM image and the corresponding elemental mapping of the CoIn_2_S_4_/rGO composite (Figure 3) revealed a uniform distribution of Co, In, S and C. The surface morphology and microstructure of pristine CoIn_2_S_4_ and as-synthesized CoIn_2_S_4_/rGO composite were further characterized by TEM. Figure 4a,b reveals the CoIn_2_S_4_ sample at various magnifications. The HRTEM images revealed that the (311) plane of CoIn_2_S_4_ was composed of prominent lattice fingers with 0.32 nm interspaces. The TEM images of the CoIn_2_S_4_/rGO composite are displayed in Figure 4c,d. The particle size of the as-synthesized CoIn_2_S_4_ was about 1 μm. Moreover, rGO was uniformly anchored with CoIn_2_S_4_. The selected area electron diffraction pattern (SAED) of the CoIn_2_S_4_/rGO sample is shown in the inset of Figure 4c. The SAED pattern revealed the halo of concentric circles. CoIn_2_S_4_ was indexed in the (311), (400), (511), and (620) planes for the various rings. We utilized BET analyses to examine the pore size distribution and specific surface area of pristine CoIn_2_S_4_ and CoIn_2_S_4_/rGO composites. Both CoIn_2_S_4_ and CoIn_2_S_4_/rGO samples exhibited the characteristic III isotherm curves of mesoporous materials.

**Figure 5:**
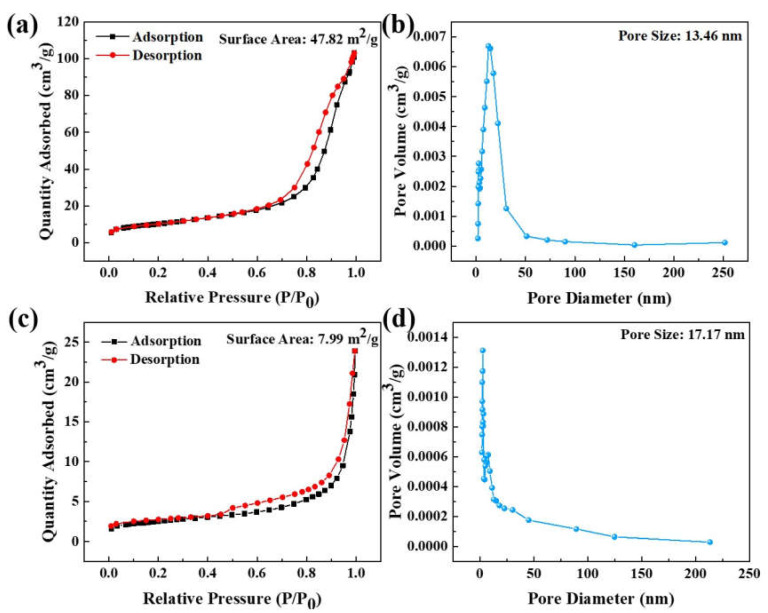
The N_2_ adsorption-desorption isotherms (**a**,**c**) and their corresponding pore size distributions (**b**,**d**) of pristine CoIn_2_S_4_ and CoIn_2_S_4_/rGO composites, respectively.

Figure 5a,c depicts the specific surface area of pristine CoIn_2_S_4_ and CoIn_2_S_4_/rGO composites. The specific surface areas of CoIn_2_S_4_ and CoIn_2_S_4_/rGO composites were 47.82 and 7.99 m^2^/g, respectively. After adding rGO, the surface area of CoIn_2_S_4_ decreased, indicating that rGO completely encapsulated the CoIn_2_S_4_ samples. The BJH method was used to calculate the pore size distributions of pristine CoIn_2_S_4_ and the CoIn_2_S_4_/rGO composite, as shown in Figure 5b,d. CoIn_2_S_4_ and CoIn_2_S_4_/rGO had BJH desorption average pore diameters of 13.46 and 17.17 nm, respectively.

**Figure 6:**
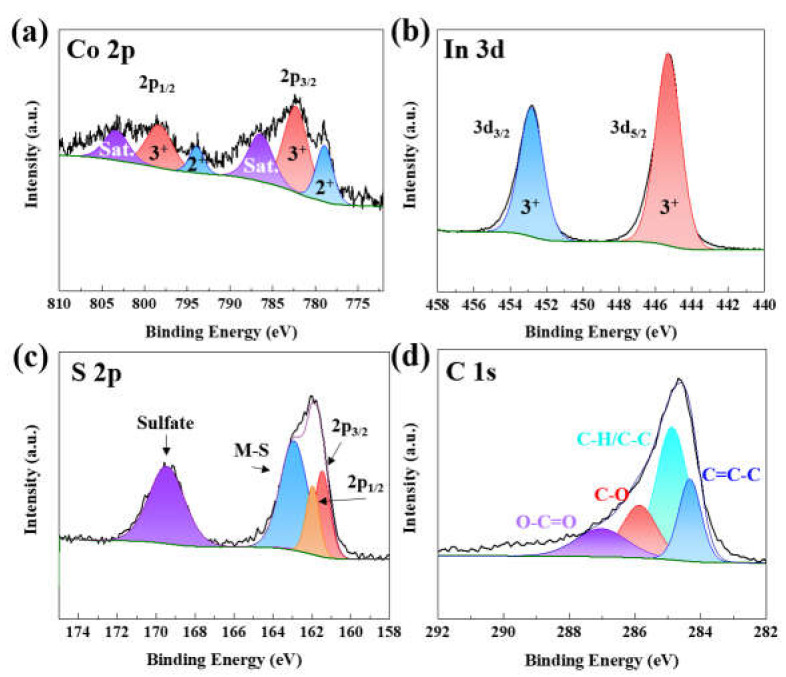
The XPS spectra of (**a**) Co 2p, (**b**) In 3d, (**c**) S 2p, and (**d**) C 1s for the CoIn_2_S_4_/rGO composite.

The chemical valence state, binding information, and surface elemental composition of the as-synthesized CoIn_2_S_4_/rGO composite were investigated using XPS. As shown in Figure 6a, the deconvolution of Co 2p_1/2_ and Co 2p_3/2_ resulted in two peaks attributable to Co^3+^ (782.9 and 798.0 eV) and Co^2+^ (778.3 and 784.3 eV), respectively. The In 3d spectrum consisted of two peaks: In 3d_3/2_ (452.3 eV) and In 3d_5/2_ (444.9 eV). As shown in Figure 6c, the spectrum of S 2p consisted of three principal peaks and a sulfate peak (169.2 eV). The peaks at 161.8 eV and 162.9 eV corresponded to the 2p_3/2_ and 2p_1/2_ spectra of S, while the peak at 163.3 eV corresponded to the sulfur-metal (S-M) bond. The peak of S-M (M=Co or In) may provide additional evidence for the formation of CoIn_2_S_4_. Figure 6d depicts the C 1s XPS peak of the CoIn_2_S_4_/rGO composite. The binding energies of 284.2, 284.8, 285.7, and 287.1 eV were associated with C=C–C, C–H/C-C, C–O, and O–C=O, respectively. Consequently, the XPS results confirmed that we successfully obtained a pure-phased CoIn_2_S_4_/rGO composite with strong chemical bonds via the hydrothermal method. Figure S1 and Table S1 show XPS survey of as-synthesized CoIn_2_S_4_/rGO composite and elemental analysis of CoIn_2_S_4_/rGO based on XPS survey, respectively. The rGO content in CoIn_2_S_4_/rGO composite was estimated to be 25.78 wt%, which was very closed to theoretical values of rGO content of 30 wt%.

**Figure 7:**
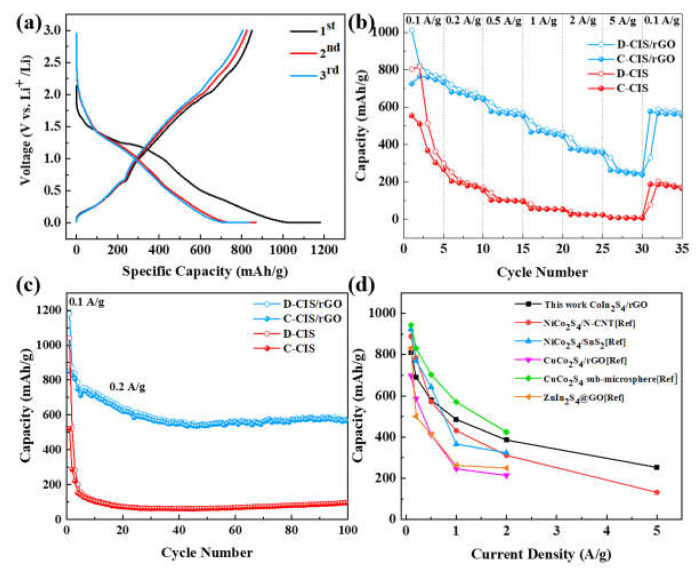
(**a**) The corresponding charge-discharge curves of CoIn_2_S_4_/rGO electrodes in the voltage range of 0.01–3.0 V; (**b**) the rate capacity of CoIn_2_S_4_ and CoIn_2_S_4_/rGO composites; (**c**) cyclic performance of pristine CoIn_2_S_4_ and CoIn_2_S_4_/rGO composites at a current density of 0.2 A/g; (**d**) rate comparison among earlier studies of bimetallic sulfide anodes for LIBs.

The charge/discharge profiles of the as-synthesized CoIn_2_S_4_/rGO electrode at 0.1 A/g for the first three cycles are depicted in Figure 7a. In the first cycle, the charge and discharge capacities of CoIn_2_S_4_/rGO were 1180 and 850 mAh/g, respectively. At the beginning of the cycle, the coulombic efficiency was 72%. However, the charge and discharge capacities of CoIn_2_S_4_/rGO were 838 and 805 mAh/g with a 96% coulombic efficiency after three cycles. The irreversible capacity fading is attributable to the irreversible decomposition of the liquid electrolyte during charging, forming an SEI layer. Figure 7b depicts the rate capability tests of pristine CoIn_2_S_4_ and CoIn_2_S_4_/rGO electrodes with varying current densities. The charge capacities of the CoIn_2_S_4_/rGO electrode were 781, 680, 574, 478, 277, and 265 mAh/g at current densities of 0.1, 0.2, 0.5, 1, 2, and 5 A/g, respectively. When the current density was switched back to 0.1 A/g, the reversible capacity of CoIn_2_S_4_/rGO was 563 mAh/g. Figure 7c depicts the cycle performance of bare CoIn_2_S_4_ and CoIn_2_S_4_/rGO composites. The initial current density used for electrode formation was 0.1A/g. Between the fourth and hundredth cycles, the current density decreased to 0.2 A/g. After 100 cycles, the pristine CoIn_2_S_4_ exhibited a capacity of 84 mAh/g. The rGO matrix buffered the dramatic volume expansion of CoIn_2_S_4_ during the lithiation and de-lithiation processes, resulting in a higher reversible capacity of 670 mAh/g for the CoIn_2_S_4_/rGO electrode.

**Figure 8:**
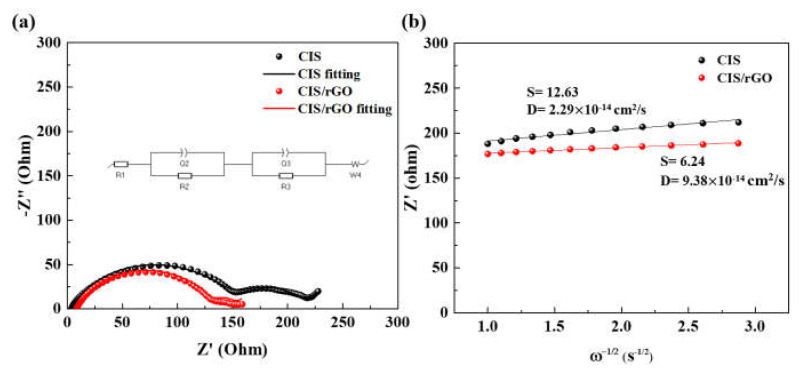
(**a**) Nyquist plots of pristine CoIn_2_S_4_ and as-synthesized CoIn_2_S_4_/rGO composite after 2.5 cycles; (**b**) relation between Z’ and ω^−1/2^ for CoIn_2_S_4_ and CoIn_2_S_4_/rGO electrode at low frequencies.

Figure 8a depicts the electrochemical impedance spectroscopy (EIS) plots of pristine CoIn_2_S_4_ and CoIn_2_S_4_/rGO composites after 2.5 cycles. The semicircle in the middle frequency range represents the solid electrolyte interphase (SEI) resistance (R_SEI_) and charge transfer resistance (R_ct_) at the electrode/electrolyte interface. The straight line in the low-frequency region is related to the diffusion resistance of lithium ions. The R_SEI_/R_CT_ values of pristine CoIn_2_S_4_ and CoIn_2_S_4_/rGO composites were 145.7/125.9 Ω and 68.3/14.0 Ω, respectively, based on the equivalent circuit fitting results. Due to the contribution of the rGO matrix, both SEI and charge transfer resistances for CoIn_2_S_4_/rGO decreased, indicating improved electronic conductivity of the electrode and enhanced electrochemical kinetics. To interpret the kinetic behavior of the electrochemical reaction, the Li^+^ diffusion coefficient (D_Li_^+^) is calculated. D_Li_^+^ can be defined as:

(1) $$D=\frac{R^{2}\;T^{2}}{2A^{2}n^{2}F^{4}C^{2}\sigma_{w}^{2}}$$

where *R* is the ideal gas constant; *T* is room temperature; *A* is the surface area of the electrode; *n* is the number of electrons; *F* is the Faraday constant; *C* is the concentration of Li^+^; *σ* is the slope of the fitting line. As a result, there is a linear relationship between Z’ and ω^−1/2^ of the pristine CoIn_2_S_4_ and the as-synthesized CoIn_2_S_4_/rGO composites. The diffusion coefficients of pristine CoIn_2_S_4_ and CoIn_2_S_4_/rGO were calculated to be 2.29 × 10^−14^ cm^2^/s and 9.38 × 10^−14^ cm^2^/s, respectively. The CoIn_2_S_4_/rGO electrode had nearly four times the D_Li_^+^ value of the pristine CoIn_2_S_4_ electrode.

**Figure 9:**
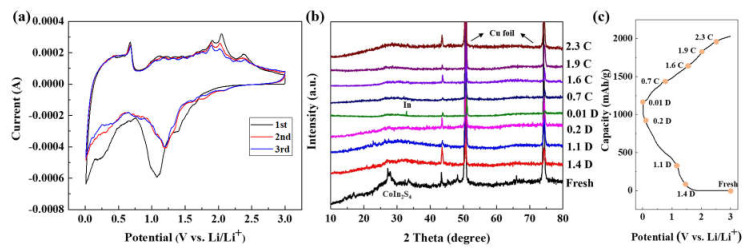
(**a**) CV curves of CoIn_2_S_4_/rGO at a scan rate of 0.1 mV/s in the first three cycles; (**b**) ex situ XRD measurements of as-synthesized CoIn_2_S_4_/rGO electrodes after different charge and discharge states; (**c**) the corresponding discharge/charge profile of CoIn_2_S_4_/rGO electrodes of potential indication at the 1st cycle.

The reduction and oxidation potentials of CoIn_2_S_4_/rGO were determined in the potential range of 0.01 V–3.0 V (V vs. Li/Li^+^) using cyclic voltammetry (CV) measurements. As demonstrated in Figure 9a, the irreversible peak at 1.1 V in the initial lithiation process is attributable to the reduction of CoIn_2_S_4_ to metal Co. [27,32,33,34] The formation of SEI during the first cathodic cycle is attributable to the 0.6 V reduction peak. The reversible peaks at 0.7, 1.6, and 1.9 V in the first anodic cycle can be ascribed to the oxidation of Co and in [28,29]. The cathodic peaks shifted to a higher potential in subsequent cycles than in the first. The activation phase can be distinguished during the initial discharge. Moreover, the CV curves of the CoIn_2_S_4_/rGO composite during the second and third lithiation/de-lithiation cycles nearly overlapped, demonstrating excellent cycling stability. To clarify the lithium reaction mechanism, ex situ XRD analyses of the CoIn_2_S_4_/rGO electrode were also performed (Figure 9b). At 28, 34, and 48°, three diffraction peaks were observed for the fresh CoIn_2_S_4_/rGO electrode, attributed to the (311), (400), and (440) planes of the CoIn_2_S_4_. After charging to 1.4 V (lithiation), the corresponding peaks of CoIn_2_S_4_ disappeared, suggesting that Co metal was produced first during the conversion of CoIn_2_S_4_. At the end of the charge stage (0.01 V), the peak at 2Θ = 33° represented the In metal diffraction peak. Next, it was demonstrated that there was no obvious peak during the subsequent lithiation. Based on ex situ XRD results, the following reaction mechanism for CoIn_2_S_4_ with Li ions was proposed:

CoIn_2_S_4_ + 8Li^+^ + 8e^−^ ↔ Co + 2In + 4Li_2_S (2)

The reversible capacity of rGO decreased with the increase in sintering temperature from ~1000 to ~200 mAh/g, which resulted from the content and types of functional groups in rGO. In this study, we used 350°C as the sintering temperature, thus, the reversible capacity of rGO-350 °C was ~1250 mAh/g. The theoretical capacity of CoIn_2_S_4_ was 514.4 mAh/g. In this study, we introduced 30% rGO in the CoIn_2_S_4_ anode. So, the reversible capacity of CoIn_2_S_4_/rGO was 1250 × 0.3 + 514.4 × 0.7 = 375 + 360.08 = 735 mAh/g. That is the reason why our CoIn_2_S_4_/rGO exceeded the theoretical capacity of CoIn_2_S_4_.

**Figure 10:**
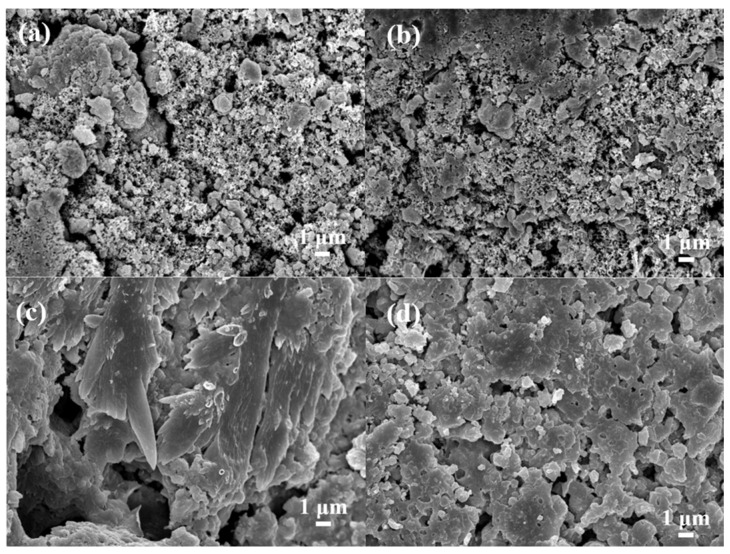
SEM images of (**a**) CoIn_2_S_4_ and (**b**) CoIn_2_S_4_/rGO electrodes before discharge/charge cycles, (**c**) CoIn_2_S_4_ and (**d**) CoIn_2_S_4_/rGO electrodes after 35 cycles.

Figure 10a,b depicts SEM images of pristine CoIn_2_S_4_ and CoIn_2_S_4_/rGO electrodes before cycling. There was no discernible distinction between the two electrodes. After 30 cycles of discharge/charge testing, the surface morphology of the pristine CoIn_2_S_4_ electrode, as depicted in Figure 10c, exhibited voids and fractures. Nevertheless, the CoIn_2_S_4_/rGO electrode (Figure 10d) maintained its compact structure and surface morphology. The ex situ SEM results indicated that rGO could prevent the cracking of the CoIn_2_S_4_ anode during the charge and discharge processes. Overall, the as-synthesized rGO-wrapped CoIn_2_S_4_ anode significantly improved the electrochemical performance. The following characteristics should be highlighted: not only can the highly electronic-conductive rGO 3D network structure provide the CoIn_2_S_4_ anode material with a batch-covered matrix that buffers severe volume changes during charging and discharging, but its porous structure also provides additional channels for Li ions during charging and discharging.

## 4. Conclusions

In summary, a novel spinel-based CoIn_2_S_4_/rGO composite anode was demonstrated by a hydrothermal reaction. The CoIn_2_S_4_/rGO composite anode provided eight times the capacity performance of the pure CoIn_2_S_4_ electrode (84 mAh/g at 0.2 A/g), with a reversible capacity of 670 mAh/g at 0.2 A/g. Furthermore, the CoIn_2_S_4_/rGO composite electrode exhibited greater cyclic and rate-specific capabilities than the pure CoIn_2_S_4_ electrode. In addition, Nyquist impedance analysis revealed that the improved performance of the CoIn_2_S_4_/rGO anode could be attributed to the incorporation of rGO, which shortens the ion diffusion pathway and prevents aggregate formation. Furthermore, ex situ XRD results revealed the Li storage mechanism of CoIn_2_S_4,_ which involves the formation of amorphous Co and In metals. The excellent electrochemical performance of CoIn_2_S_4_/rGO makes it a promising anode for LIBs.

## Data Availability

Data that support the findings of this study are included within the article.

## Supplementary Materials

The following supporting information can be downloaded at: https://www.mdpi.com/article/10.3390/nano12244367/s1, Figure S1: XPS survey of as-synthesized CoIn_2_S_4_/rGO composite; Table S1: Elemental analysis of CoIn_2_S_4_/rGO based on XPS survey.
