# Supplementary material for: Reduced Graphene Oxide-Wrapped Novel CoIn2S4 Spinel Composite Anode Materials for Li-ion Batteries
Source: Nanomaterials (Basel). 2022 Dec 7;12(24):4367. doi: 10.3390/nano12244367 (PMC9781618; doi:10.3390/nano12244367)
Supplement: Supplementary file 1 [file nanomaterials-12-04367-s001.zip › nanomaterials-2072225-supplementary.pdf]

Supporting Information

Reduced graphene oxide-wrapped novel $CoIn_2S_4$ spinel composite anode materials for Li-ion batteries

Ting-Yu Lee and Wei-Ren Liu*

Department of Chemical Engineering, R&D Center for Membrane Technology, Center for Circular Economy, Chung Yuan Christian University, 200 Chung Pei Road, Chung Li District, Taoyuan City 32023, Taiwan

**Correspondence: wrliu1203@gmail.com; Tel.: +886-3-2653315; Fax: 886-3-2653399*

.

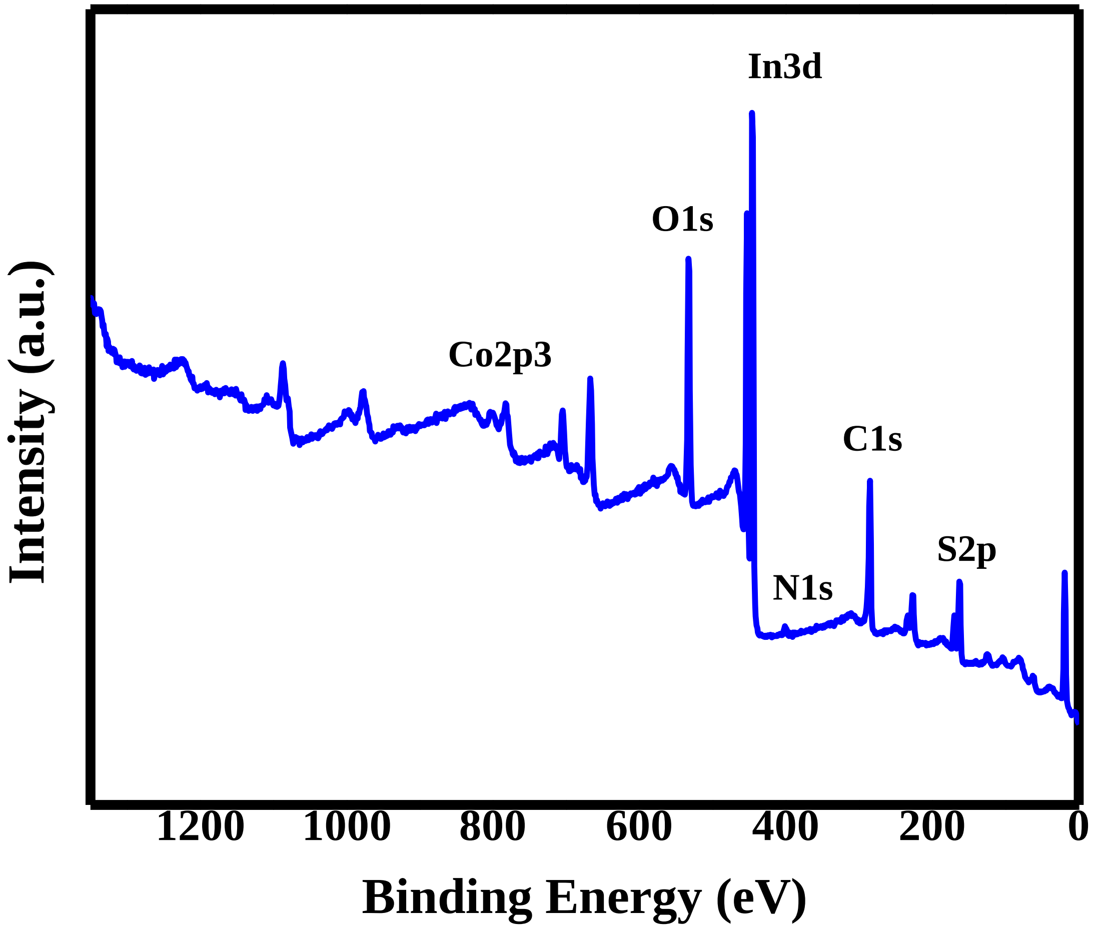

Figure S1. XPS survey of as-synthesized $CoIn_2S_4$/rGO composite

Table S1. Elemental analysis of $CoIn_2S_4$/rGO based on XPS survey.

Element	O1s	In3d	C1s	S2p	Co2p3
Atomic(%)	20.90	7.54	54.25	14.20	3.74
Weight(%)	13.24	34.29	25.78	18.00	8.73
